# Supplementary material for: Longitudinal Assessment of Serum 25-Hydroxyvitamin D Levels during Pregnancy and Postpartum—Are the Current Recommendations for Supplementation Sufficient?
Source: Nutrients. 2023 Jan 10;15(2):339. doi: 10.3390/nu15020339 (PMC9863354; doi:10.3390/nu15020339)
Supplement: Supplementary file 1 [file nutrients-15-00339-s001.zip › nutrients-2140094-supplementary.pdf]

nutrients

Supplementary Table S1 Pregnancy outcomes in singleton and twin pregnancies

	singleton pregnancies n=182	twin pregnancies n=49	p-value
GA at delivery in weeks, median (IQR)	39.29 (38.29-40.29)	36.86 (35.86-37.43)	<0.001
Live birth, n (%)	181 (99.5)	48 (98)	0.136
Stillbirth, n (%)	0 (0)	1 (2)	
Neonatal death <1 week, (%)	1 (0.5)	0 (0)	
Small for gestational age infant 1, n (%)	24 (13.4)	18 (37.5)	<0.001
Small for gestational age infant 2, n (%)		15 (31.3)	
Mode of delivery			<0.001
Spontaneous, n (%)	92 (50.8)	2 (4.1)	
Elective caesarean, n (%)	3 (1.7)	1 (2)	
Primary caesarean, n (%)	51 (28.2)	36 (73.5)	
Secondary caesarean, n (%)	17 (9.4)	10 (2.4)	
Ventouse, n (%)	15 (8.3)	0 (0)	
Emergency caesarean, n (%)	3 (1.7)	0 (0)	
Preterm birth, n (%)	17 (9.3)	27 (55.1)	<0.001
HDP, n (%)	8 (4.4)	4 (8.2)	0.29
GDM, n (%)	17 (9.3)	4 (8.2)	0.81
IGDM, n (%)	15 (8.2)	3 (6.1)	0.63
PROM, n (%)	19 (10.4)	4 (8.2)	0.64
Preterm labour, n (%)	9 (4.9)	6 (12.2)	0.06
Cervical Insufficiency, n (%)	4 (2.2)	5 (10.2)	0.010
Pregnancy complications, n (%)	47 (25.8)	24 (48)	0.003
NICU admission infant 1, n (%)	8 (4.4)	8 (16.3)	0.004
RDS infant 1, n (%)	9 (4.9)	9 (18.4)	0.002
NICU admission infant 2, n (%)		10 (20.8)	
RDS infant 2, n (%)		9 (18.8)	

Supplementary Table S2 Vitamin D deficiency in the first trimester and association with complications in twin pregnancies presented as absolute frequencies and percentages in parenthesis

	Vitamin D <50 nmol/l n=23	Vitamin D >50 nmol/l n= 26	p-value
Preterm birth, n (%)	12 (52.2)	15 (57.7)	0.70
HDP, n (%)	1 (4.3)	3 (11.5)	0.36
GDM, n (%)	2 (8.7)	2 (7.7)	0.90
IGDM, n (%)	0 (0.0)	3 (11.5)	0.09
PROM, n (%)	2 (8.7)	2 (7.7)	0.90
PTL, n (%)	3 (13.0)	3 (11.5)	0.87
Cervical Insufficiency, n (%)	3 (13.0)	2 (7.7)	0.54
Pregnancy complications, n (%)	11 (45.8)	13 (15.0)	0.77
NICU admission infant 1, n (%)	3 (13.0)	5 (19.2)	0.56
RDS infant 1, n (%)	4 (17.4)	5 (19.2)	0.87
SGA infant 1, n (%)	11 (47.8)	7 (28.0)	0.156
NICU admission infant 2, n (%)	4 (17.4)	6 (24.0)	0.573
RDS infant 2, n (%)	3 (13.0)	6 (24.0)	0.331
SGA infant 2, n (%)	9 (39.1)	6 (24.0)	0.259
